# “First Do No Harm”. No-Fault Compensation Program for COVID-19 Vaccines as Feasibility and Wisdom of a Policy Instrument to Mitigate Vaccine Hesitancy

**DOI:** 10.3390/vaccines9101116

**Published:** 2021-09-30

**Authors:** Stefano D’Errico, Martina Zanon, Monica Concato, Michela Peruch, Matteo Scopetti, Paola Frati, Vittorio Fineschi

**Affiliations:** 1 Department of Medical, Surgical, and Health Science, University of Trieste, Strada di Fiume 44, 34149 Trieste, Italy; sderrico@units.it (S.D.); martina.zanon@virgilio.it (M.Z.); monica.concato@studenti.units.it (M.C.); michela.peruch95@gmail.com (M.P.); 2 Department of Anatomical, Histological, Forensic and Orthopaedic Sciences, Sapienza University of Rome, Viale Regina Elena 336, 00185 Rome, Italy; matteo.scopetti@uniroma1.it (M.S.); paola.frati@uniroma1.it (P.F.); 3 IRCCS Neuromed, Via Atinense 18, 86077 Pozzilli, Italy

**Keywords:** vaccination, COVID-19, no-fault compensation, adverse events, solidarity, vaccine hesitancy

## Abstract

Vaccines are so far proven to be safe, although related adverse events cannot be excluded. The urgency for COVID-19 vaccines determined a dilution of the general expectations of safety and efficacy of vaccination (from safe and effective to safe and effective enough). In many countries, a no-fault program was established to compensate individuals who experienced serious vaccine-related injuries. The impressive number of administrations worldwide and the legal indemnity afforded to manufacturers of approved vaccines that cannot be pursued for compensation fed the debate about the availability of a compensation model for COVID-19 vaccine-related injuries. Several European countries have long introduced a system, Vaccine Injury Compensation Programs, to compensate people who suffer physical harm because of vaccination. In Europe, COVID-19 vaccination is strongly recommended for the general population and in many states is declared mandatory for healthcare workers. In 1992, Italy edited Law no. 210 providing legal protection for individuals who reported injuries after mandatory and recommended vaccinations as a no-fault alternative to the traditional tort system. Despite its recommended nature, COVID-19 vaccination is excluded from the no-fault model in several European states, and the Italian government is called to provide clear and firm instructions for the management of the many requests for compensation. The authors provide an overview of the existing compensation models in Europe and analyse available legislative proposals.

## 1. Introduction

Despite their high safety profile, vaccine-related adverse events are reported, though rarely. The low risk is counterbalanced by the benefits of widespread immunization, with immunization programs estimated to save 2 to 3 million lives globally each year [1]. Vaccine injury compensation programs (VICPs) are no-fault schemes established to compensate individuals who experience serious vaccine-related harm. As indicated by the term “no-fault”, VICPs do not require injured parties or their legal representatives to prove negligence or fault by the vaccine provider, health care system, or the manufacturer before compensation. They seek to waive the need for accessing compensation for vaccine-related harms through litigation, where processes are lengthy and clear negligence can be difficult to prove. Under a no-fault VICP, governments compensate individuals harmed by properly manufactured vaccines with the intention of removing the need for individuals to use legal or other processes against manufacturers [2]. Globally, 25 jurisdictions have VICPs. Most of these countries are in Europe, and a recent review study identified 16 nations in Europe with VICPs [3]. Debates around vaccine injury compensation programs have arguably taken on increased significance in the context of the COVID-19 pandemic, as billions of COVID-19 vaccines are intended for global administration, and manufacturers of approved COVID-19 vaccines are being afforded legal indemnity through purchase agreements, which means that they cannot be pursued for compensation for vaccine-related harm [4].

## 2. Worldwide Compensation Models for COVID-19 Vaccine-Related Adverse Events

Up to 27 July 2021, the European Centre for Disease Prevention and Control (ECDC) estimated a total of 442,802,569 doses of COVID-19 vaccines administered in EU/EEA countries, with 53.7% of the adult population over 18 years old (199,148,427) fully vaccinated and 68.5% (253,643,470) vaccinated with one dose [5]. According to the ECDC reports, up to June 2021, 0.2% of cases of suspected adverse reactions after COVID-19 vaccination were reported to EudraVigilance [6]. Most suspected adverse reactions reported so far relate to general reactions and the administration site (flu-like illness, headache, pain at the application site, chills, fatigue, nausea, fever, dizziness, weakness) occurring within two days of vaccination and almost all within seven days [7]. Major adverse events were represented by anaphylaxis (2 to 5 people per million vaccinated), myocarditis and pericarditis, and Guillain Barré syndrome. Few cases of thrombosis with thrombocytopenia syndrome (TTS) were also reported following the administration of non-replicating viral vector COVID-19 vaccines [8,9,10,11,12,13,14,15,16,17,18]. Reports of death after COVID-19 vaccination are rare and limited to single reports [19]. More than 339 million doses of COVID-19 vaccines were administered in the United States from 14 December 2020 through to 19 July 2021. During this time, the Vaccine Adverse Event Reporting System (VAERS) received 6207 reports of death (0.0018%) among people who received a COVID-19 vaccine.

The debate around the importance to compensate for vaccine-related injuries has taken on more pertinence as COVID-19 vaccine programmes are rolled out worldwide [20]. In February 2021, the World Health Organization (WHO) signed an agreement on behalf of the COVAX Facility for the administration of a no-fault compensation program for the 92 low and middle-income countries and economies eligible for support via the Gavi COVAX Advance Market Commitment (AMC) of the COVAX Facility, to support COVID-19 vaccination and to ensure globally equal access to compensation for vaccine harms [21]. It represents in actuality the first and only vaccine injury compensation mechanism operating on an international scale and offers a fast and transparent process to receive compensation for rare but serious adverse events associated with COVAX vaccines until 30 June 2022. In high-income countries, few existing compensation mechanisms incorporate side effects of the COVID-19 vaccines, based on the declared health emergency states and the incentives of a wide vaccination campaign. In other cases, the existing no-fault compensation programs for routine immunization do not incorporate COVID-19 vaccination adverse events. In the following discussion, the main features of the compensatory regimes of some countries are described extensively or in summary (Table 1) to provide an overview and at the same time focus attention on some peculiar regulatory aspects.

### 2.1. Australia

Vaccination for COVID-19 is voluntary in Australia. Until 2021, Australia did not have a no-fault vaccine damage compensation scheme. In June 2021, the National Conference held by the Public Health Association of Australia (PHAA) confirmed the introduction of a no-fault Vaccine Injury Compensation Scheme for COVID-19 vaccines administered in Australia [22].

### 2.2. Austria

COVID-19 vaccination is not obligatory in Austria. The government representatives have repeatedly underlined that there will be no compulsory vaccination. Austrian law provides for a public-law system for the payment of compensation by the state under the Vaccine Damage Act [23]. The state will pay compensation in the event of damage to a person’s health resulting from the administration of certain vaccines. Compensation is granted based on a request for social security against the state under the Vaccine Damage Act. The claim is assessed in an administrative proceeding. COVID-19 vaccinations have been included into the above-mentioned Regulation on Recommended Vaccinations (Verordnung der Bundesministerin für Gesundheit und Frauen über empfohlene Impfungen).

### 2.3. Belgium

A COVID-19 vaccination is not mandatory, and there is currently no legislation about COVID-19 vaccine compensation.

### 2.4. Canada

In August 2021, the Canadian government announced it would require COVID-19 vaccination for federal public service employees and members of the military. As of June 2021, the Canadian government launched a national vaccine injury compensation program for people who experienced severe adverse reactions to an approved COVID-19 vaccine [24]. The pan-Canadian Vaccine Injury Support Program (VISP) provides financial support to those determined to have experienced a serious and permanent injury after receiving a Health Canada-authorized COVID-19 vaccine in Canada on or after 8 December 2020. According to the Public Health Agency of Canada (PHAC), financial support will also be available to the dependents of those who have died after receiving a vaccination. That support includes income replacement, payment for injuries, death benefits including funeral expenses, and other eligible costs, such as uncovered medical expenses. The amount of financial support provided will be determined on a case-by-case basis, but compensation will be retroactive from the date of the injury or death. The PHAC stated that it will compensate life-threatening or life-altering injuries that require in-person hospitalization or prolongation of existing hospitalization and result in persistent or significant disability or incapability or whether the outcome is a congenital malformation or death.

### 2.5. China

COVID-19 vaccination is not mandatory, and the compensation program is regulated according to the existing legislation for both compulsory and not compulsory vaccinations [25]. The State implements a vaccination-related abnormal reaction compensation system. Relevant compensation will be paid in the case of any in-vaccination or post-vaccination death, severe disability, or damage, such as organ tissue injury to a recipient that is identified as (or cannot be ruled out as being) a vaccination-related abnormal reaction. Compensation scope, standards, and procedures for vaccination-related abnormal reactions will be prescribed by the State Council, and specific implementing measures will be formulated by provincial governments, autonomous regions, and municipalities directly under the central government. Compensation costs incurred about vaccinations with immunization program vaccines (i.e., compulsory vaccinations) will be paid out of vaccination funds as arranged by competent departments of finance under the relevant provincial governments, autonomous regions, or municipalities directly under the central government. On the contrary, compensation costs incurred about vaccinations with non-immunization program vaccines (i.e., non-compulsory vaccination) will be borne by the pertinent vaccine marketing authorization holders.

### 2.6. France

In France, COVID-19 vaccination is not mandatory, but the French government has recommended COVID-19 vaccinations for certain categories of individuals, such as seniors in retirement homes, professionals working in these retirement homes at risk of developing a severe form of COVID-19, healthcare professionals aged 50 years or over, disabled people, or persons suffering from chronic illness. Since 18 January 2021, the government has recommended vaccinations for seniors aged 75 years or over and vulnerable patients with a high risk in case of contamination. The existing compensation program includes compensation for injuries related to compulsory vaccinations only. There is no special procedure for compensation of damages resulting from recommended non-compulsory vaccinations. A specific compensation procedure is applicable in the context of the COVID-19 vaccination campaign. The patient can lodge a claim directly with the National Office for Compensation of Medical Accident (ONIAM) and will not have to prove a defect with the product or any fault by the healthcare professional to receive compensation. Any person who has suffered damage as a result of the COVID-19 vaccination program is eligible for compensation pursuant to the general principles of French civil law, since this vaccine is non-compulsory [26].

### 2.7. Germany

COVID-19 vaccination is not mandatory in Germany, and compensation for damage due to COVID-19 vaccines is covered under existing legislation. In particular, Germany has a no-fault compensation program in place that also applies to COVID-19 vaccinations as long as they are publicly recommended as stated in Section 60 of the German Infection Protection Act [27]. The no-fault compensation program applies to compulsory vaccination and to non-compulsory vaccinations as long as the vaccination is publicly recommended. The compensation is a flat-rate scheme influenced by various factors, depending primarily on the individual degree of injury.

### 2.8. Hong Kong

In Hong Kong, the government set up the COVID-19 Vaccination Programme (“Programme”), which allows Hong Kong residents to be voluntarily vaccinated free of charge. In February 2021, the Hong Kong government introduced the Expert Committee on Clinical Events Assessment following COVID-19 Immunization including members with expertise in paediatrics, neurology, pharmacology, haematology, immunology, pharmacy, forensic pathology, and microbiology and discussed the indemnity fund for adverse events following immunization with COVID-19 vaccines with an initial fund size of 1 billion dollars to provide support to individuals who have proof of suffering adverse events associated with anti-COVID-19 vaccination [28]. It has been proposed that the affected individual will have access to the fund resources if there is certification by a registered medical practitioner of the adverse event and if the evaluation outcome of the Expert Committee cannot rule out that the event is not associated with the administration of a vaccine under the Government’s COVID-19 Vaccination Program. The claims should be presented within two years from the last dose of the vaccine, and the time limit could be reviewed nearer the time, having regard to the pattern and timing of occurrence of listed events.

### 2.9. Netherlands

COVID-19 vaccination is not mandatory, nor are other vaccinations, so all compensation is related to non-compulsory vaccinations. The general Dutch legal system for compensation of damages, codified in article 95 and in book 6 of the Dutch Civil Code applies to any claims of damages caused by COVID-19 vaccines. There is no specific or applicable regulation in place [29].

### 2.10. Portugal

In Portugal, a COVID-19 vaccination is not obligatory, but Portugal has implemented a national plan for COVID-19 vaccination. Currently, there is no specific legislation governing vaccine compensation. Thus, possible compensation claims are regulated by the Portuguese Civil Code and by Law no. 67/2007 concerning the civil liability of the state and legal persons governed by Public Law. No specific legislation has been enacted regarding COVID-19 vaccines [30].

### 2.11. Singapore

COVID-19 vaccine is voluntary in Singapore, and there are currently no specific laws or regulations governing vaccine compensation, save for the Vaccine Injury Financial Assistance Programme (VIFAP) introduced by the Singapore Ministry of Health (MOH) and funded by the Singapore government [31]. Only Singapore citizens and residents who have received the COVID-19 vaccination in Singapore through the government program and who are seriously injured after the COVID-19 vaccination are eligible for financial assistance under the VIFAP. Compensation in the form of financial support up to certain limits under the VIFAP will be provided in cases of hospitalization, which requires care in a High Dependency or Intensive Care Unit, death, or permanent severe disability. Those who experience serious side effects can also continue to concurrently receive support through applicable government healthcare schemes and subsidies.

### 2.12. South Africa

COVID-19 vaccination is still voluntary in South Africa. On 24 February 2021, it was announced that the government would set up a no-fault compensation fund to cover claims related to serious injuries caused by the COVID-19 vaccine [32]. The actual legal framework surrounding this compensation fund has not yet been released. However, the Health Minister of South Africa stated in January 2021 that any person who voluntarily chooses to get the vaccine will be required to sign an indemnity waiver, indemnifying the individual from any liability stemming from any potential risk and harm caused by the vaccine. The validity of such a waiver would need to be considered should the compensation scheme be rolled out. Furthermore, the waiver will need to be tested against existing consumer protection legislation. In addition, the WHO has announced the establishment of the vaccine injury compensation program, which is a no-fault compensation system that will be available to 92 low and middle-income countries, including South Africa.

### 2.13. South Korea

Vaccination against COVID-19 is freewill in South Korea. The South Korean Disease Control and Prevention Agency (KCDC) announced that it will expand compensation coverage for those suffering from severe side effects after getting a COVID-19 vaccine [33]. The compensation will provide up to 10 million KRW for medical expenses. The Korean government has implemented a system for providing compensation to those falling severely ill or dying due to COVID-19 vaccines.

### 2.14. Sweden

All vaccinations are voluntary in Sweden, including vaccination against COVID-19. The Swedish Government has submitted a proposal on a new law on state compensation for personal injuries caused by COVID-19 vaccines as a complement to the financial protection offered by the Swedish Pharmaceutical Insurance [34]. The Swedish Pharmaceutical Insurance enables individuals suffering from adverse effects of pharmaceutical treatments (including vaccines) to receive compensation without having to undergo legal proceedings. Insurance, which is no-fault, is usually a more favourable, simpler, and less expensive way for an individual to receive compensation than going to court. Regarding potential serial injuries caused by COVID-19 vaccinations, there is an even greater risk that the limitation for serial injuries under the Insurance will become applicable. Consequently, the Swedish government has submitted a proposal for a law on state compensation for personal injuries that may arise as a result of the COVID-19 vaccination. According to the proposal, state compensation shall also be paid for personal injuries caused by a COVID-19 vaccine manufactured by a pharmaceutical company that is not a shareholder in the Insurance. The new law is proposed to enter into force on 1 December 2021 and shall also apply to COVID-19 vaccine injuries that may have occurred before that date. Pending the entering into force of the proposed law, the Swedish Legal, Financial, and Administrative Services Agency (Sw. Kammarkollegiet) has signed an agreement with LFF Service AB on settlement of claims and compensation for vaccine injuries caused by COVID-19 vaccines that are not included in the Insurance.

### 2.15. United Kingdom

In the UK, COVID-19 vaccination is not mandatory, and as of 31 December 2020, people injured after COVID-19 vaccination can access financial assistance through the Vaccine Damage Payments Scheme (VDPS), as occurred before with HPV, meningitis B, and H1N1 (swine flu) [35]. Generally, only those who were administered vaccines as part of a childhood immunization program are covered under the VDPS. However, because COVID-19 vaccines will be rolled out to a large proportion of the adult population, the government amended the eligibility requirements, ensuring adults who are administered a COVID-19 vaccine in the UK or as part of an armed forces medical treatment will be covered by the scheme too. The VDPS is a safety net to help ease the burden on individuals who have in extremely rare circumstances experienced harm due to receiving a government-recommended vaccine. It is not a compensation scheme. Rather, it provides a one-off, tax-free lump sum—currently £120,000—for those suffering a severe disability as a result of a vaccine against a disease listed under the Vaccine Damage Payments Act [36]. The main weakness of the scheme is that there is an upper limit of £12,000,030, and so awards will often be much less than tort law damages for similar harm.

### 2.16. United States

COVID-19 vaccination is not mandatory in the United States. The declaration of the health emergency state, by the Department of Health and Human Services since 2020, resulted in the exclusion of COVID-19 vaccine injuries from the existing Vaccine Injury Compensation Program (VICP), which ensures quick and fair compensation for vaccine injuries and insulates manufacturers from liability [37]. This declaration triggered the Public Readiness and Emergency Preparedness (PREP Act) that established that all people injured by vaccines given as countermeasures during a declared emergency bring claims under only the Countermeasures Injury Compensation Program (CICP), which is less generous and less accessible than the VICP [38]. People who are vaccinated during the declared public health emergency who reported adverse events with COVID-19 vaccines could be compensated only if severely injured and have limited awards for damages. Compensation for pain, suffering, or emotional distress are excluded, and a high burden of proof is required. It has also been observed that the process for compensation of COVID-19 vaccine-related injuries is more difficult than for other vaccines included in the VICP and more expensive because reimbursement for attorneys’ fees is not available. Van Tassel et al. proposed that any FDA-approved COVID-19 vaccine should fall under the VICP as soon as possible as should all future vaccines targeting active pandemics. It has also been suggested that to prevent inequities in access to compensation, lawmakers could establish that all vaccines recommended by the CDC to ameliorate a public health emergency must be immediately added to the VICP and Congress should require that a 75-cent excise tax be applied to all vaccine for pandemic viruses in the United States, which is already done for childhood vaccines to finance the Vaccine Injury Compensation Trust Fund.

## 3. COVID-19 Vaccine-Related Adverse Events Compensation: An Italian First?

Italy is one of the European countries to have been hit hardest by COVID-19, with over 4.0 million cases and 128,000 (3.2%) deaths [39]. COVID-19 vaccination was introduced in Italy in December 2020 as not compulsory but highly recommended vaccination. In application of the Strategic Plan on vaccines approved by parliament on 2 December 2020, the free administration of vaccines to the entire population has begun, according to an order of priority, which considers the risk of disease, the types of vaccines, and their availability. The vaccination campaign began with the inoculations of the following groups: health and social care workers, residents and staff of residential care facilities for the elderly, and person of advance age or considered “fragile” for comorbidities. All costs of COVID-19 vaccination are borne by the state. Italy is also the first country in Europe to have made the COVID-19 vaccine mandatory for healthcare professionals and to have stated that the healthcare worker who refuses vaccination should be employed in tasks that do not risk spreading the virus or be suspended without salary for up to one year [40]. Recently, the Italian government imposed restrictions on unvaccinated people because of the aim to contain a resurgence of infections and to provide the largest vaccine population coverage within September 2021 [41]. With the same decree, doctors who administered COVID-19 vaccine were protected from liability as long as the shot had been carried out in accordance with the Ministry of Health’s instructions. As of 4 August 2021, against a total of 65,926,591 doses administered, 84,322 reports of adverse events following COVID-19 immunization have been made on the Italian Pharmacovigilance network; there was a reporting rate of 128 per 100,000 doses (mostly minor adverse events), with a reporting rate of serious adverse events of 16 per 100,000 doses, mean age of 49 years, and a prevalence of onset (80.2%) within 48 h of vaccination [42]. In the young population (12 to 19 years old) 530 adverse events were reported, with a reporting rate of 27 per 100,000 doses. Many adverse events occurred in the first 48 h (60%) or in the first week (18%) or in the following weeks (19%). A total of 423 reports of death occurring from two hours to a maximum of 78 days from COVID-19 vaccine have been reported. In 244 of the cases, death was recorded after the first dose, and in 127 after the second. In most cases the death was related to previous pathologies, and anaphylactic shock or major allergic reactions were excluded. In 59.6% of the cases, causality assessment performed with the WHO algorithm excluded the relationship between death and COVID-19 vaccine; in 33.6%, the causality was undetermined, and in 4.2%, unclassifiable due to lack of information necessary for the application of the algorithm. Correlation between death and COVID-19 vaccination was reported in 2.6% of the cases.

In 1992, with Law no. 210, the Italian government stated that whoever has received, because of vaccination required by the law or by order of an Italian health authority, injury or illness, from which a permanent physical–psychological impairment is derived, could obtain an indemnity by the State [43]. The Constitutional Court with judgment of 16 April 2012 no. 107 declared the constitutional illegitimacy of article 1, paragraph 1, extending the compensation to vaccinations that are not mandatory but rather highly recommended so as to avoid disparity among individuals exposing themselves to risk also for the benefit of protecting the community at large (e.g., vaccination for measles, mumps and rubella, or influenza) [44,45]. Pursuant to constant case law and according to law, the benefits allowed are considered a social security benefit and are independent of any requirement to demonstrate the fault. Therefore, a victim remains free to seek full compensation in tort law. This possibility was confirmed a few years later by Law no. 229/2005. Injuries related with not compulsory COVID-19 vaccination cannot be compensated according to the existing no-fault compensation scheme. In July 2021, local health authorities asked the Health Ministry for clear indications for the management of requests of compensation of people injured after COVID-19 vaccines and to establish a common pathway for all citizens to exclude inequities in the access to compensation. It has been observed that the Constitutional Court ruled out on the obligation of the state to recognize the indemnity also in some cases, in which the vaccine, although not mandatory, was strongly recommended by the Italian government as for COVID-19 vaccination. However, it is also well known that it is not possible to draw a principle of general application for all cases in which the vaccine is recommended by the government.

## 4. Discussion

Vaccines are the most important public health intervention to prevent the spread of infectious diseases. Since the identification of the SARS-CoV-2 virus, the scientific community has provided an exceptional effort in the development of over 300 vaccine projects. A few of these new vaccines were approved for emergency use to start one of the widest vaccination campaigns ever. The results of COVID-19 vaccination are encouraging worldwide, indicating that a COVID-19 vaccine is safe and produces a good immune response [46,47,48]. Despite their safety and efficacy, people of various nations still revealed hesitancy towards COVID-19 vaccination. In the past, it has been observed that the fear of adverse events and the lack of information on immunization are the main factors of vaccine hesitancy [49]. The rapid pace of vaccine development and misinformation in popular and social media contributed in these months to the lack of confidence in COVID-19 vaccination and complacency of the population about vaccination, leading to delay and refusal of vaccination, despite its availability, and undermining the success of coronavirus disease vaccination programs [50].

No-fault compensation schemes are the most common instrument used by governments to protect all those who are damaged by compulsory or recommended vaccinations, and governments’ efforts should be addressed to including all people injured after the COVID-19 vaccination as a hopeful strategy for the decline of vaccine hesitancy.

A 2020 global review reported 25 World Health Organization (WHO) jurisdictions had no-fault compensation vaccine injury compensation programs (16 in the European region, 6 in Asia, 2 in America, 1 in Oceania) [51]. Most of the existing programs are implemented at the central or federal government level and are government-funded. In 65% of cases, the vaccine injury compensation program covers injuries arising from vaccines that are registered in the country; in 78% of jurisdictions, claim and decision-making processes are purely administrative rather than involving civil litigation, and standards of proof between vaccination and injury are required. In 52% of jurisdictions, compensation covers medical expenses, disability pensions, and death benefits; all these payments are usually based on the severity of the injury. In 44% of cases, standardized compensation is provided, as in the UK where a sum lump payment of 140.000 euro is offered [52]. Economic losses, pain, suffering, and emotional stress could be compensated only in a few compensation models [53]. Furthermore, in 65%, the right to seek compensation was guaranteed both through civil litigation and through a compensation plan, although not at the same time. In 26% of jurisdictions, damages could be sought only through a compensation scheme.

In Italy, since 1992, with Law no. 210, the lawmaker provided legal protection and the provision of economic indemnity for all people who reported injuries after mandatory or recommended vaccinations as a no-fault alternative to the traditional tort system in order to compensate vaccine-related injuries [54]. The law refers to all damages suffered by subjects due to compulsory vaccination by law or order of a health authority, unvaccinated subjects who have suffered a permanent impairment as a result of contact with a vaccinated person, subjects who for reasons of work or assignment of their office, or in order to access a foreign country, have undergone vaccinations that, although not mandatory, were necessary, subjects at risk who are working in hospital health facilities who have undergone vaccinations even if vaccinations are not compulsory for them [55]. With respect to both compulsory and non-compulsory vaccinations, those individuals who can demonstrate that they suffered an injury because of the vaccination will instead be eligible for compensation. Both in the cases of compulsory and non-compulsory vaccinations, material and non-material damages can be compensated. Compensation may cover the economic burden for the cure of the injuries, permanent damages, and death. The indemnity paid in case of damage from compulsory vaccination under Law 210 of 1992 is an all-inclusive sum that is paid out for the purpose of social solidarity, and a cap on compensation is not provided [56].

The debate around the importance to compensate for vaccine-related injuries has taken on more pertinence as COVID-19 vaccine programs have been rolled out worldwide [57]. In February 2021, the World Health Organization (WHO) signed an agreement on behalf of the COVAX Facility for the administration of a no-fault compensation program for the 92 low and middle-income countries and economies eligible for support via the Gavi COVAX Advance Market Commitment (AMC) of the COVAX Facility, to support COVID-19 vaccination and to ensure globally equal access to compensation for vaccine harms [58]. Actually, it represents the first and only vaccine injury compensation mechanism operating on an international scale and offers a fast and transparent process to receive compensation for rare but serious adverse events associated with COVAX vaccines until 30 June 2022.

Looking across the political landscape, several general observations can be ventured at this point. There are three different approaches to addressing COVID-19 vaccine injury globally: patients with adverse events may bear the costs associated with their injuries; they may seek to be made whole through litigation against private-sector actors (principally manufacturers), or they may be compensated through publicly supported systems that draw from public-sector and private-sector contributions; the third approach, a no-fault compensation system for adverse events attributed to COVID-19 vaccination, seems to balance the utilitarian principle funding the social contract supporting immunization and the principle of integrity and dignity of the individual.

Under a no-fault vaccine injury compensation system, all the governments involved with COVID-19 vaccination may compensate individuals who are harmed by properly manufactured COVID-19 vaccines instead of requiring them to use legal or other processes against manufacturers.

A no-fault compensation scheme answers the ethical issue that a community that promotes immunization, knowing individuals could be injured, must share the burden of the cost of injuries.

It has been observed that the success of the COVID-19 vaccine campaign will also depend on widespread public trust in the safety of the vaccine (or vaccines), the diligence of the manufacturers and health workers producing and distributing them, and the adequacy of legal safeguards to prevent and compensate for inadvertent harm. Public acceptance of the vaccine can be facilitated by guaranteeing the right to compensation that covers not only health costs but also the loss of livelihoods.

Maintaining public trust in vaccines despite scepticism will depend on effective public health messaging, transparent regulatory decision-making, and effective communication about the guarantee that victims of adverse effects are rapidly and adequately compensated.

As a result, there is a strong ethical requirement for adequate compensation at the occurrence of severe side-effects. The citizen or health care professional who has been vaccinated, primarily for the benefit of others and of society should not be exposed to potentially life-changing side-effects without compensation. This view follows both from general considerations of fairness, and from more specific considerations concerning how society ought to reciprocate when its members suffer significant harm while engaging in activities that provide societal benefits and that are encouraged by society.

Although the effect of a no-fault compensation system by improving or decreasing public confidence in vaccines is uncertain, efforts need to be provided to strengthen information to the whole population to support no-fault compensation programs in cases of anti-COVID-19 vaccination injuries to promote vaccination [59]. A lack of public awareness has been observed about the existence of vaccine injury compensation programs worldwide. At the same time, strict standards of proof that immunization caused injury need to be shared with the scientific community since the early phases of the compensation program as a guarantee for equity of access of population.

Setting up a coordination mechanism or recommendations on who should provide compensation for the side-effects of the COVID-19 vaccine and in what manner it should be provided must be considered firstly. The risk is that citizens living in countries in which a no-fault compensation model is provided will have different access to compensation even though they have been vaccinated with the same vaccine.

The scheme should be based on a no-fault model, and as a basic principle, compensation should be automatically made available where a COVID-19 vaccine has been found to cause harm without the need for the patient to establish and prove a defect in the product or fault of the manufacturer. All those situations in which the vaccination has been associated with a particular adverse effect should be classified to presume a logical sequence of cause and effect and establish a proximate temporal relationship between the vaccination and the injury. The quantum of financial redress of any award should be fair and equitable and be close enough to the level of damages awarded before the courts to dissuade resort by those injured to litigation, but the existence of the scheme should not affect the availability of litigation through the courts. The compensation should be free to access by any affected patients, and funding provision and support should be made available to affected patients to assist them in making applications and fully quantifying their current and future needs and past losses [60,61]. The possibility to be compensated after injuries should be widely communicated as the importance to be vaccinated, with proper public outreach and dissemination of information regarding the scheme to the general public.

The compensation system should be simple, quick, and accessible, but above all no-fault, to provide just and equitable damages. There should be no arbitrary cap on damages. Proving causation could be facilitated by an expert-led process allowing for the identification of situations in which vaccination is linked to a particular adverse effect.

Being proactive in establishing such a fund will improve the chances of any immunization program being effective while reducing overall costs to society.

The potential costs of this policy with COVID-19 vaccination remain questionable. As regards costs, previous experiences with vaccine-related injuries compensation suggests that doubts of sustainability are unsubstantiated because the costs tend to be manageable and predictable.

In the meantime, more transparency in communication about compensation procedures for vaccine side effects is necessary for all those countries in which the compensation for COVID-19 vaccine-related adverse events is not in actuality foreseen [62].

As has been said, it is now widely accepted that patient safety should be a key factor in health policy, and there is widespread acceptance of the proposition that a risk management strategy and a no-fault compensation system for post-vaccinal adverse events may be important factors in supporting and providing incentives for safety improvement. There is a particular focus on initiatives that promote disclosure of adverse events [63]. In this regard, administrative compensation proposals need to grow as a politically and socially viable idea around which the demonstration of patient safety benefits can be built [63].

## 5. Conclusions

In order to provide reassurance to those administered COVID-19 vaccines, it is our view that a COVID-19 vaccine compensation regime should be created without delay in Italy. To avoid expending resources on the costs of the litigation system, it would be preferable to divert victims to a compensation fund specifically created for adverse effects caused by COVID-19 vaccines. Such a regime should be based on a no-fault model, with a simple, swift, and accessible procedure, providing fair compensation to those who have suffered an adverse effect. Compensation should be based on need and the sums awarded should be high enough to avoid disputes to supplement the premium. Behavioural science suggests such schemes will only be attractive if they represent a better bargain than gambling on the uncertain outcome of litigation. Being proactive in establishing such a fund will improve the chances of any immunization programme being effective and at the same time reduce overall costs to society. It also fits in with a philosophy that prefers to nudge citizens to make the choices the state wants rather than imposing a system and removing existing rights.

## Figures and Tables

**Table 1:** COVID-19 vaccine compensation regimes in different countries.

Country	Obligatoriness	Costs	Compensation	Coverage	Damage	Requirements	Compensation Criterion
Austria	No	Free	Specific procedure	Full	All	Causality	Individual assessment (pensions) and tariffs (care allowance)
Belgium	No	Free	Existing legislation	Certain categories	All	Causality	Individual assessment
China	No	Free	Existing legislation	Full	All	Reference catalogue	Individual assessment
France	No	Free	Specific procedure	Full	All	Causality	Individual assessment
Germany	No	Free	Existing legislation	Full	All	Causality	Individual assessment (pharmaceutical company) and tariffs (no-fault programme for physicians)
Italy	No	Free	Existing legislation	Full	All	Causality	Individual assessment (compensations) and tariffs (indemnities)
Netherlands	No	Free	Existing legislation	Citizenship and age over 18	All	Causality	Individual assessment
Peru	No	Free	Specific procedure	Full	All	Causality	Undefined
Poland	No	Free	Existing legislation	Full	All	Causality	Individual assessment
Portugal	No	Free	Existing legislation	Full	All	Causality	Individual assessment
Russia	No (restrictions for the unvaccinated)	Free	Existing legislation	Full	All	Causality	Individual assessment
Singapore	No	Free	Existing legislation	Citizenship and residence	Severe and death	Causality	Individual assessment
South Africa	No	Free	Not regulated	Not determined	Not known	Not established	Not known
United Kingdom	No	Free	Existing legislation	Full	Severe and death	Causality	Tariffs

## Data Availability

The data used to support the findings of this study are available from the corresponding author upon request.
